# Editorial: Developing Successful Neuroprotective Treatments for TBI: Translational Approaches, Novel Directions, Opportunities and Challenges

**DOI:** 10.3389/fneur.2019.01326

**Published:** 2019-12-17

**Authors:** Stefania Mondello, Anwarul Hasan, Deborah A. Shear

**Affiliations:** ^1^Department of Biomedical and Dental Sciences and Morphofunctional Imaging, University of Messina, Messina, Italy; ^2^Department of Mechanical and Industrial Engineering, College of Engineering, Qatar University, Doha, Qatar; ^3^Brain Trauma Neuroprotection Branch, Center for Military Psychiatry and Neuroscience, Walter Reed Army Institute of Research, Silver Spring, MD, United States

**Keywords:** traumatic brain injury (TBI), neuroprotection, therapy, translational research, drug development, clinical trial design, mesenchymal stem cells, phenotyping

Traumatic brain injury (TBI) constitutes a critical health problem. More than 50 million new cases occur worldwide each year, accounting for upwards of a million deaths (roughly 2,700 deaths per day) and a global financial burden of $US400 billion (1, 2). Overall, TBI-related disability is increasing globally, negatively affecting families and society, as well as health-care systems and economies (2). Among survivors, nearly half of those with moderate or severe TBI require years of intensive therapy and face substantial functional impairment and reduced life expectancy (3); however, also mild TBIs and concussions can lead to persistent adverse outcomes.

Despite thousands of preclinical studies in laboratory animals and hundreds of randomized controlled clinical trials (RCTs) testing neuroprotection approaches with different pathophysiological targets (4, 5), to date, no intervention has demonstrated to be unequivocal effective and improve the long-term functional outcome following TBI (1, 6).

This book incorporates reviews and research articles from leaders in the field describing promising therapeutic avenues, which span the spectra of TBI severity and have undergone experimental and clinical investigation. What emerges is a panoramic view of a field actively exploring alternative strategies and novel directions, such as preclinical-drug screening multimodel multispecies consortia (Kochanek et al.) (7, 8), capable to tackle the challenge of heterogeneity in TBI.

Recognizing the critical neuronal loss and wide variability existing in pathophysiologic mechanisms triggered by TBI, the current focus is on the regenerative potential and pleiotropic neuroprotective properties of stem cell therapy for TBI (9, 10). Accordingly, diverse authors have provided perspectives with respect to the application of mesenchymal stromal cells (MSCs) (Carbonara et al.) and the anti-inflammatory role of neural stem cell transplantation (Kassi et al.), and also generated new compelling data (Nasser et al.).

Sophisticated experiments reinterpreting previous unsuccessful therapeutic interventions (11, 12), including erythropoietin (Robinson et al.), hypothermia (Szczygielski et al.), and selective brain cooling (Leung et al.) have also been conducted providing authors with the opportunity to identify potential reasons for clinical failure. In this analysis, a complex array of factors, comprising a lack of precise diagnose and endophenotype characterization, inadequate measures of outcomes, and tools to inform tailored and individualized treatment emerges as main barriers to advance clinical care in TBI (13). Complementing this conceptual framework, a strong emphasis has also been placed by several contributors on the need for a mechanistic approach to guide drug development. This strategy can better inform target selection and streamline the hard translational decision process. As a consequence, investigations on mitochondrial dysfunction (Pandya et al.), microglial activation (Madathil et al.), cerebral microcirculation impairment (Bellapart et al.), and gut dysbiosis (Rice et al.) following TBI are presented, highlighting their directly involvement in disease pathogenesis and outcome, and role as therapeutic target candidates.

Finally, the volume includes experimental and human studies which add further evidence for the use of neuroimaging (Bajaj et al.) and biofluid markers (Bhatti et al. and DeDominicis et al.) as surrogate endpoints of clinical benefit and treatment response after therapeutic intervention in TBI, supporting their incorporation in future clinical trials (14). Imaging and biofluid markers can, in fact, be instrumental in identifying and characterizing pathophysiologic mechanisms leading to more accurate and finer-grained disease classification which, in turn, may be used to enrich or stratify patient groups, to demonstrate target engagement, and/or as proof of treatment efficacy (14–16). These factors can play a transformative role in designing effective clinical trials, increasing treatment effectiveness as well as reducing healthcare costs (16–18).

Overall, this volume is intended to provide novel perspectives and insights into the critical research area of TBI treatment, foster knowledge and innovation in the arena of drug development, and stimulates new frameworks and testable hypotheses that can help inform and refine the next generation of TBI clinical trials.

We thank our colleagues for devoting their time, efforts, and clinical and scientific experience. This book would not have been possible without their important contributions. We also thank the editorial team for their dedicated support and assistance. Last, and most important, we are indebted to all patients with TBI and their families for their invaluable contributions. They represent our greatest source of inspiration and all our endeavors are directed toward improving their outcome and quality of life.

## Author Contributions

SM wrote the original draft, assembled and incorporated comments from the co-authors, and crafted the final draft. All of the other co-authors contributed to manuscript review and revision.

### Conflict of Interest

The authors declare that the research was conducted in the absence of any commercial or financial relationships that could be construed as a potential conflict of interest.
